# The social evolution of sleep: sex differences, intragenomic conflicts and clinical pathologies

**DOI:** 10.1098/rspb.2018.2188

**Published:** 2019-01-02

**Authors:** Gonçalo S. Faria, Susana A. M. Varela, Andy Gardner

**Affiliations:** 1 School of Biology, University of St Andrews, Dyers Brae, St Andrews KY16 9TH, UK; 2 Instituto Gulbenkian de Ciência, 6 Rua da Quinta Grande, 2780-156 Oeiras, Portugal; 3 cE3c – Centre for Ecology, Evolution and Environmental Changes, Faculdade de Ciências, Universidade de Lisboa, Campo Grande, 1749-016 Lisboa, Portugal

**Keywords:** inclusive fitness, intragenomic conflict, genomic imprinting, kin selection, sexual selection, sleep disorders

## Abstract

Sleep appears to be essential for most animals, including humans. Accordingly, individuals who sacrifice sleep are expected to incur costs and so should only be evolutionarily favoured to do this when these costs are offset by other benefits. For instance, a social group might benefit from having some level of wakefulness during the sleeping period if this guards against possible threats. Alternatively, individuals might sacrifice sleep in order to gain an advantage over mate competitors. Here, we perform a theoretical analysis of the social evolutionary pressures that drive investment into sleep versus wakefulness. Specifically, we: investigate how relatedness between social partners may modulate sleeping strategies, depending upon whether sleep sacrifice is selfish or altruistic; determine the conditions under which the sexes are favoured to adopt different sleeping strategies; identify the potential for intragenomic conflict between maternal-origin versus paternal-origin genes regarding an individual's sleeping behaviour; translate this conflict into novel and readily testable predictions concerning patterns of gene expression; and explore the concomitant effects of different kinds of mutations, epimutations, and uniparental disomies in relation to sleep disorders and other clinical pathologies. Our aim is to provide a theoretical framework for future empirical data and stimulate further research on this neglected topic.

## 1. Introduction

Sleep—defined as a reversible state of behavioural inactivity, elevated arousal threshold, and homeostatic regulation [1,2]—has been found to occur in all animal species that have been adequately studied [3,4]. Several non-exclusive hypotheses have been offered as to the biological function of sleep, including energy allocation into physiological activities that cannot be performed during the day, adaptive inactivity when activity is costly, metabolite clearance from the brain, maturation of the nervous system during ontogeny, memory consolidation, and synaptic homeostasis [5] (electronic supplementary material, table S1).

Given the apparently important benefits of sleep, individuals who sacrifice sleep would be expected to incur significant costs and, indeed, lack of sleep is known to cause or exacerbate a very wide range of health problems, ranging from cardiovascular diseases [6] and type 2 diabetes [7] to psychological distress [8] and cancer [9]. From an evolutionary perspective, sacrifice of sleep will only have been favoured provided that there are substantial compensating benefits. For instance, a social group may benefit from having its members waking up at different times throughout their sleeping period if this helps to protect the group from potential dangers [10,11]. Alternatively, individuals might sacrifice sleep to gain an advantage over their mate competitors [12–14]. In both of these scenarios, individuals who give up opportunities to sleep may have an important impact on the survival and mating success of their social partners, making sleep an important aspect of an individual's social behaviour.

However, at a fundamental level, the social evolutionary pressures that have shaped investment into sleep versus wakefulness remain entirely obscure. It is not even clear whether sacrificing sleep is a relatively altruistic activity—incurring costs to the individual and yielding benefits to social partners—or relatively selfish—yielding benefits to the individual at a cost to their social partners. Moreover, the distinction between altruistic versus selfish sleep sacrifice could potentially explain sex differences in sleep schedules and modulate conflicts of interest between an individual's maternal- and paternal-origin genes over the individual's investment into sleep versus wakefulness. Such intragenomic conflict would be expected to underpin a range of medical disorders and pathologies in relation to the biology of sleep.

Here, we investigate under which circumstances individuals are favoured to invest more time into sleep versus wakefulness, with a focus on individuals' social environment, and how that may affect gene expression and explain sleep pathologies. Methodologically, we use a personal fitness approach to kin selection [15,16], the results of which analysis may be interpreted in terms of inclusive fitness [17]. First, we analyse how an individual's sleep schedule may be modulated by the degree of genetic relatedness to their social partners, providing a contrast between altruistic versus selfish sacrifice of sleep. Second, we explore the possibility for sex-specific social evolutionary pressures—arising from sex differences in relatedness between social partners and the associated benefits of sleep sacrifice—to drive sex-specific sleeping schedules. Third, we investigate whether there is potential for intragenomic conflict to occur between an individual's maternal-origin versus paternal-origin genes over the investment that the individual makes into sleep versus wakefulness. Fourth, we use the ‘loudest voice prevails’ principle [18] to translate such conflict into readily testable predictions concerning patterns of gene expression, specifically ‘genomic imprinting’. Finally, we show that these patterns of gene expression lead to readily testable predictions concerning the effects of different kinds of mutations, epimutations, and uniparental disomies on sleep disorders and other pathologies. As these predictions relate largely to data that remain to be collected, our overall aim is to provide a theoretical framework for future empirical work and to stimulate research activity on this neglected topic.

## 2. Is sleep selfish or altruistic?

Natural selection favours those individuals that pass on more copies of their genes to future generations [19,20]. According to the theory of inclusive fitness, an individual may achieve this either by increasing their own reproductive success (direct fitness) or by increasing the reproductive success of other individuals with whom they share genes in common (indirect fitness) [15,21]. Hamilton's rule [15,21–23] provides an encapsulation of this logic: a social behaviour will be favoured by natural selection so long as *−C* + *Br* > 0, where *C* is the loss of reproductive success incurred by the actor, *B* is the gain in reproductive success by the actor's social partners, and *r* is the genetic relatedness of the actor to their social partners. This is an extremely general result, that holds irrespective of whether the genetical trait varies in a continuous or discontinuous manner, whether selection is weak or strong, whether genes interact additively or nonadditively, and so on (reviewed by [17]). If the social behaviour stabilizes at an intermediate evolutionary optimum then this must occur when the direct and indirect fitness effects exactly cancel each other out (−*C* *+* *Br* = 0; note that this is true even if the cost and benefit of the social behaviour change over evolutionary time). This means that the behaviour must be either altruistic (*C* > 0 and *B* > 0) or selfish (*C* < 0 and *B* < 0) at equilibrium [15,24,25].

**Figure 1.**
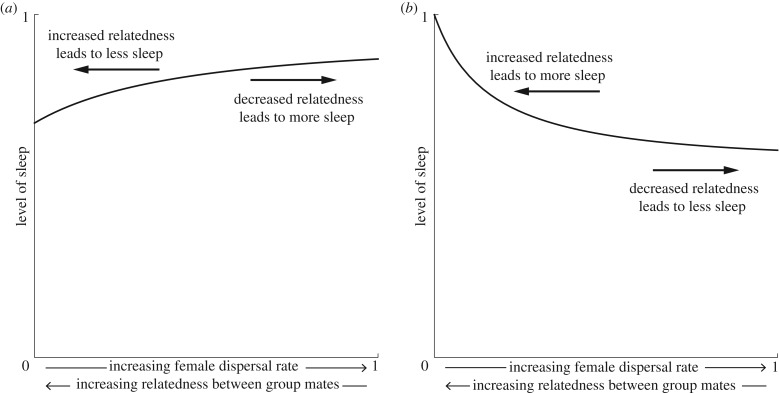
How much an individual should sleep depends on the relatedness between the individuals in a group. When individuals sacrifice sleep in order to remain alert to dangers which may befall the group (*a*), individuals sacrifice more sleep when relatedness is higher, which is the case when female dispersal is lower. When individuals sacrifice sleep in order to gain an advantage over their mate competitors (*b*), individuals sacrifice more sleep when relatedness is lower, which is the case when female dispersal is higher. The following parameter values were used for both panels: male dispersal rate *d*_m_ = 0; budding dispersal rate *d*_B_ = 1; number of adult females *n*_f_ = 4; number of adult males *n*_m_ = 4; minimum level of sleep *m* = 0.05; and benefits of sleeping throughout the night *b*_f_ = *b*_m_ = 1. Additionally, in (*a*) the level of a threat is *a* = 1 and the mating opportunities that females and males can obtain through sleep sacrifice is *c*_f_ = *c*_m_ = 0, while in (*b*) the level of a threat is *a* = 0 and the mating opportunities that females and males can obtain through sleep sacrifice is *c*_f_ = *c*_m_ = 1. Here, we consider female-biased dispersal—see electronic supplementary material, figure S1 for male-biased dispersal.

Consider an individual who is genetically predisposed to having relatively more sleep. If this leads to an increase in their own reproductive success (*C* < 0) and a decrease in their social partners' reproductive success (*B* < 0), then this sleep strategy may be described as selfish and, conversely, individuals who tend to sacrifice sleep may be described as behaving altruistically. An example of such a scenario is when individuals may choose to sacrifice sleep in order to protect their group from threats during the night, such as surprise attacks from other groups or predators. The genetic relatedness of group mates then determines how much sleep an individual should be favoured to sacrifice in order to protect their group from such dangers. To illustrate such a scenario, we incorporate between-group dispersal into Haldane's [26] classic ‘tribe splitting’ model of human altruism (see electronic supplementary material for details), revealing that a higher rate of dispersal of individuals between groups, which leads to lower relatedness among group mates, incentivises individuals to devote more time to sleep (figure 1*a* and electronic supplementary material, figure S1*a*).

Conversely, if an individual who sleeps relatively more thereby incurs a loss of reproductive success (*C* > 0) and provides a benefit to their social partners (*B* > 0), then they can be said to be behaving altruistically and, conversely, an individual who has a tendency to sacrifice sleep is behaving selfishly. An example of such a scenario is when individuals may choose to sacrifice sleep in order to pursue mating opportunities, and thereby reduce mating opportunities for their social partners. Again, relatedness between group mates is expected to modulate how much time an individual should devote to sleep in such a scenario, but in the opposite direction from before. Turning again to the tribe-splitting model for an illustration, we reveal that as the rate of individual dispersal increases—and, consequently, the degree of relatedness among group members decreases—individuals are favoured to sleep less (figure 1*b* and electronic supplementary material, figure S1*b*).

So is sleeping selfish, or is it altruistic? This question remains to be answered due to a lack of scientific investigation on how genetic relatedness modulates sleep. With respect to the above scenarios, several studies have shown how predation [27–37] and sexual competition [12–14] modulate the sleep schedule of several species. Moreover, Capellini *et al*. [38] report that total duration of sleep across mammals is lower when individuals are more likely to sleep in a group, which they interpreted as either due to individuals being able to sleep more deeply—and hence not requiring such long periods of sleep—or alternatively owing to time invested in social interaction leaving less time for sleep. In humans, selfish personality traits have been shown to correlate with late sleep onset [39] and short-term mating success [40]. To our knowledge, the potential of genetic relatedness to modulate sleep in all of those scenarios remains to be investigated, and a comparative approach—taken across populations or species—may provide a more definitive means of assessing whether sleeping more is a selfish or an altruistic behaviour.

## 3. Sex differences in sleeping behaviour

Above we have shown that sleep is expected to be modulated by its impact on the reproductive success of the individual and the individual's social partners. However, the components of direct (−*C*) and indirect (*Br*) fitness are liable to be different for females and males, and this suggests that females and males may be favoured to adopt different sleep schedules.

**Figure 2.**
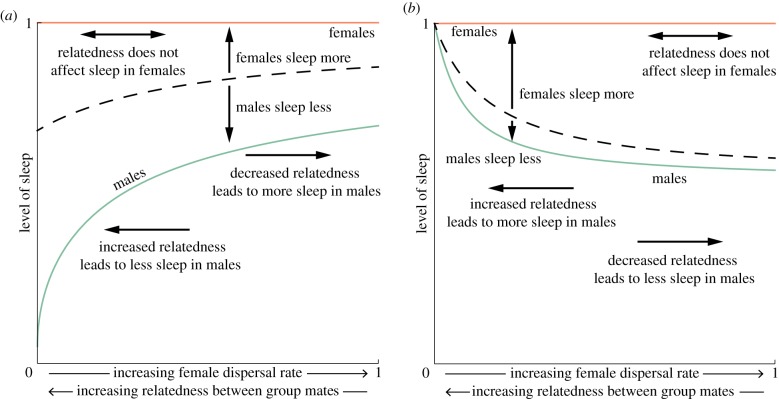
Females and males may be favoured to have different sleeping levels. Given that females are less related to their social partners than males, females favour more sleep when (*a*) the sleep sacrifice is being used to protect the group against threats. When (*b*) sleep sacrifice is being used to increase male reproductive success, females do not favour any sleep sacrifice, with males being the only ones to sacrifice sleep to gain an advantage over their mate competitors. Dashed line represents the favoured level of sleep when this is constrained to be the same for females and males. The following parameter values were used for both panels: male dispersal rate *d*_m_ = 0; budding dispersal rate *d*_B_ = 1; number of adult females *n*_f_ = 4; number of adult males *n*_m_ = 4; minimum level of sleep *m* = 0.05; and benefits of sleeping throughout the night *b*_f_ = *b*_m_ = 1. Additionally, in (*a*) the level of a threat is *a* = 1 and the mating opportunities that females and males can obtain through sleep sacrifice is *c*_f_ = *c*_m_ = 0, while in (*b*) the level of a threat is *a* = 0 and the mating opportunities that females and males can obtain through sleep sacrifice is *c*_f_ = 0 and *c*_m_ = 1, respectively. Here, we consider female-biased dispersal—see electronic supplementary material, figure S2 for male-biased dispersal.

If individuals sacrifice sleep in order to protect their group against night-time dangers, then there is no obvious reason to suspect that this should incur different costs (*C*) or provides different benefits (*B*) to their social partners. Nevertheless, females and males might differ with respect to how genetically related they are to their group mates (*r*_f_ ≠ *r*_m_), and this alone could drive sex differences in sleeping habits. Ancestral human populations may have been characterized by female-biased dispersal [41], which would have led to females and males being differently related to their group mates. Returning to the tribe-splitting model for the purpose of illustration (see electronic supplementary material for details), we show that female-biased dispersal—which leads to females being less related to their group mates than are males (*r*_f_ < *r*_m_)—leads to females being favoured to invest more in sleep than are males, when sleep sacrifice is altruistic (figure 2*a* and electronic supplementary material, figure S2*a*).

But females and males do differ in many aspects of their biology, particularly in relation to reproduction. Females often invest more time and energy into raising their offspring than males who, not having this limitation, are free to pursue additional mating opportunities with females that remain available [42]. In this sense, females may be seen as a resource for which males have to compete [43], such that sexual selection usually acts more strongly in relation to males. Insofar as these differences in fitness components are relevant to the evolution of sleep, we might expect that these, too, could favour sex-specific sleep patterns. For example, if sleep sacrifice is associated with increased mating opportunities [12] which could offset the costs associated with sleep sacrifice, then males are expected to gain more from sleep sacrifice than are females (*C*_m_ < *C*_f_). In addition, relatedness is expected to modulate how competitive the males should be. Returning to the tribe-splitting model for illustration (see electronic supplementary material for details), we show that an increased rate of individual dispersal—which reduces genetic relatedness between social partners—leads to more selfishness on the part of males and, therefore, less sleep (figure 2*b* and electronic supplementary material, figure S2*b*).

Sex-specific sleeping patterns have been reported in several non-human animal species. Specifically, some studies report that total sleep length is higher for females (in pectoral sandpipers [12], in great tits [13], and in blue tits [44]) while others report that total sleep length is higher for males (in fruit flies [45], and in mice [46]). In humans, women have been suggested to enjoy better-quality sleep [47–49]. Men are also more likely than women to perform normally during the day with less sleep [50,51], suggesting that in our evolutionary past either: (i) women have been favoured to have more sleep and men have evolved adaptations to reduce the harmful effects of less sleep, or (ii) women need more sleep than men due to basic physiological differences. Regardless, the role of relatedness in modulating any of these patterns in humans or any other animal species has not, to our knowledge, been explored empirically.

## 4. Intragenomic conflict, genomic imprinting, and sleep

The genes within an individual do not necessarily agree on how their carrier should interact with social partners. Because an individual can be more related to social partners through one parent than the other, then genes inherited from each of the two parents may differ with regards to the social behaviour that they favour [18,52,53]. Insofar as genetic relatedness is relevant to the evolution of sleeping patterns, maternal-origin genes and paternal-origin genes may then disagree on how much an individual should sleep.

**Figure 3.**
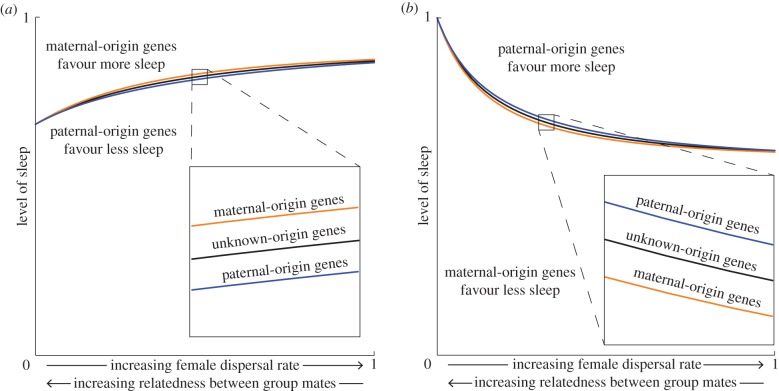
Maternal- and paternal-origin genes disagree regarding how much the individual should sleep. Maternal-origin genes (orange) and paternal-origin genes (blue) will disagree on how much an individual should sleep, which depends upon whether individuals are sacrificing sleep to (*a*) protect the group against threats or (*b*) gain an advantage over their mate competitors (with black being the level favoured by a gene ignorant of its origin). Specifically, given that relatedness is higher for paternal-origin genes, maternal-origin genes favour more sleep and paternal-origin genes less sleep if sleep is selfish (*a*). On the contrary, if sleep is altruistic, then maternal-origin genes favour less sleep and paternal-origin genes more sleep (*b*). The following parameter values were used for both panels: male dispersal rate *d*_m_ = 0; budding dispersal rate *d*_B_ = 1; number of adult females *n*_f_ = 4; number of adult males *n*_m_ = 4; minimum level of sleep *m* = 0.05; and benefits of sleeping throughout the night *b*_f_ = *b*_m_ = 1. Additionally, in (*a*) the level of a threat is *a* = 1 and the mating opportunities that females and males can obtain through sleep sacrifice is *c*_f_ = *c*_m_ = 0, while in (*b*) the level of a threat is *a* = 0 and the mating opportunities that females and males can obtain through sleep sacrifice is *c*_f_ = *c*_m_ = 1. Here, we consider female-biased dispersal—see electronic supplementary material, figure S3 for male-biased dispersal.

If individuals altruistically sacrifice sleep in order to protect their group mates from danger, then we expect that the genes for which relatedness between social partners is higher will be more strongly favoured to sacrifice their carriers sleep. Using again the tribe-splitting model as an illustration (see electronic supplementary material for details), increasingly female-biased dispersal—which reduces genetic relatedness between social partners with respect to their maternal-origin genes—leads to paternal-origin genes favouring less sleep and maternal-origin genes favouring more sleep (figure 3*a*; electronic supplementary material, figure S3*a* for the opposite pattern when dispersal is male-biased). In contrast, if individuals sacrifice sleep to increase their mating success, then the genes for which relatedness is higher will favour more sleep. Going back to the tribe-splitting model (see electronic supplementary material for details), increasingly female-biased dispersal, leads to paternal-origin genes favouring more sleep and maternal-origin genes favouring less sleep (figure 3*b*; electronic supplementary material, figure S3*b* for the opposite pattern when dispersal is male-biased). These scenarios describe what is known as intragenomic conflicts [18,52,53].

**Figure 4.**
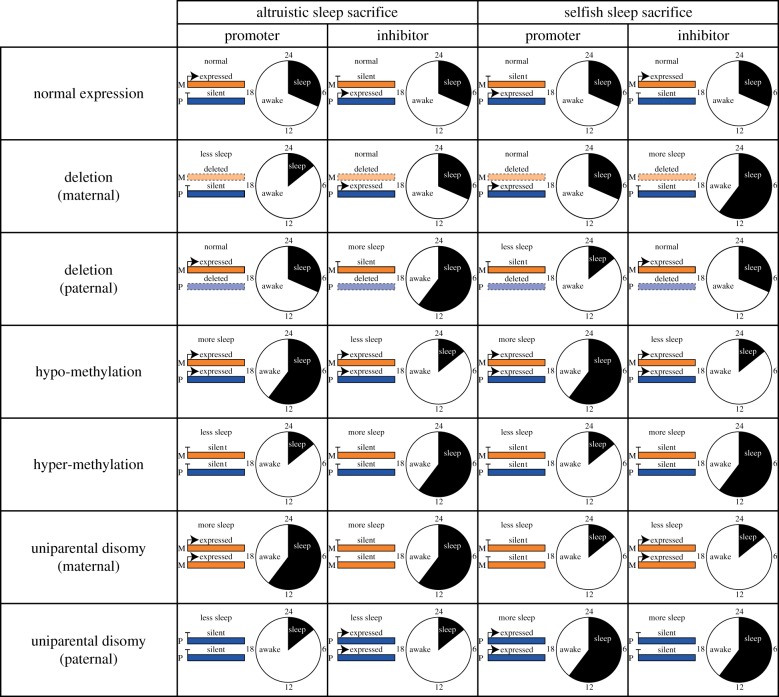
Genomic imprinting of genes responsible for level of sleep and the effects of possible disruptions. Predictions as to which gene is expressed and which gene is silent—maternal-origin gene (M, orange) or paternal-origin genes (P, blue)—when individuals are sacrificing sleep to protect the group against threats (altruism) or to gain an advantage over their mate competitors (selfishness). We consider an example for a gene that promotes sleep (promoter) and an example for a gene that inhibits sleep (inhibitor). In both cases, we assume female-biased dispersal—see electronic supplementary material, figure S4 for male-biased dispersal. Note that for simplicity we assume methylation is associated with gene silencing, as is usually the case in mammals [54]. In cases where methylation is associated with gene activation the outcome for hypo-methylation is expected to be that shown here for hyper-methylation, and vice versa.

These intragenomic conflicts are predicted to lead to parent-of-origin-specific gene expression—i.e. ‘genomic imprinting’ [18]. Consider a locus for which increased gene expression leads to more sleep—a ‘sleep promoter’. The gene that favours more sleep can get closer to its optimal level of sleep by increasing its own expression. The gene that favours less sleep, in contrast, gets closer to its optimal level of sleep by decreasing its genetic expression. Such changes continue until the gene favouring a lower level of sleep ends up silencing itself, with the gene favouring a higher level of sleep winning the intralocus conflict and, accordingly, setting the level of expression to its own optimum [18] (figure 4; see [55] for a simulation illustration). The logic is reversed for a locus in which increased gene expression leads to less sleep, a ‘sleep inhibitor’. In that case, it is the gene that favours lower level of sleep that wins the conflict, and the other gene is silenced (figure 4).

In recent years, there has been a growing interest in the genetic [56–59] and epigenetic [60–66] control of sleep. Epigenetic control comprises any molecular mechanism that changes how genes are expressed without affecting the DNA sequence itself [67] and includes genomic imprinting, which is usually described as involving methylation of the gene's regulatory regions [18]. Several genes involved in the control of sleep have been shown to be imprinted [68–76], but theoretical explanations for such patterns are relatively lacking. The only exception is Haig's [77] study of an intragenomic conflict regarding sleep, where night waking to suckle in newborns is predicted to lead to more maternal care [78,79]. In such scenarios, paternal-origin genes favour more night waking and maternal-origin genes less night waking because mothers might have future offspring from different fathers [77]. Here, we have shown that social conflicts in adults may also be relevant for the evolution of genomic imprinting in genes controlling sleep.

## 5. Sleep disorders and genomic imprinting

Genomic imprinting renders individuals functionally haploid at affected loci, and therefore vulnerable to the effects of mutations that would otherwise have been (at least partially) recessive under standard diploid gene expression [80]. These mutations are predicted to result in extreme pathologies [81]. More generally, possible kinds of mutations that can lead to dramatic consequences are: deletions, where a gene is removed from the genome; epimutations, where disruptions occur in the machinery responsible for determining the methylated pattern of a gene; and uniparental disomy, where individuals carry two copies of a maternal- or paternal-origin gene, instead of one of each. In each of these types of perturbations, specific predictions can be made about their consequences at the phenotypical level which are dependent on the selective force that led to individuals sacrificing sleep (figure 4). Conversely, if the phenotypic effect of the mutation is known, then these predictions may be used to infer whether sleep sacrifice is relatively selfish or altruistic (figure 4).

Among the most well-known examples of human pathologies that emerge from a disruption of genomic imprinting patterns are those associated with Prader–Willi and Angelman syndromes, which are hypothesized to have been evolutionarily driven by an intragenomic conflict between maternal-origin and paternal-origin genes in young children with respect to their demand of maternal resources [82]. Because a mother's future offspring might have different fathers, the child's paternal-origin genes favour greater greediness while the maternal-origin genes favour less greediness. Consequently, maternal duplication/paternal deletion of the chromosomic region 15q11-13 results in children with a phenotype that is the result of reduced maternal investment during pregnancy, such as reduced weight, or that result in reduced maternal investment in the newborn, such as poor suckling response, weak cry, and physical inactivity. In contrast, paternal duplications/maternal deletion of the chromosomic region 15q11-13 results in children with a phenotype that is the result of increased maternal care during pregnancy, such as increased weight, or that result in increased maternal care in the new-born, such as prolonged suckling response and physical hyperactivity. Sleep is also affected [68,69,71,75] because night waking in children is predicted to lead to more maternal care [78,79]. Prader–Willi syndrome is then characterized by less night waking to suckle, while Angelman syndrome is characterized by the opposite pattern, with more night waking to suckle [68,69,71,75,77]. While the nature of the conflict is different from what we explore in our analysis, it illustrates how genomic imprinting can affect sleep.

Other disorders have been hypothesized as being associated with genomic imprinting patterns, such as autism and psychopathic disorders [83,84]. Such disorders are considered to be extremes from a phenotypic-continuum, with autism being a ‘hyper-altruistic’ brain disorder (low cognitive empathy but high emotional empathy; [85]) and psychopathic disorders a ‘hyper-selfish’ brain (high cognitive empathy but low emotional empathy; [86]). The intragenomic conflict between maternal-origin genes and paternal-origin genes is over how altruistic an individual should be to their social partners. Therefore, if indeed female-biased dispersal was prevalent throughout human evolution, then relatedness would have been higher for paternal-origin genes and lower for maternal-origin genes. Accordingly, autism would be the result of paternally expressed genes and psychopathy the result of maternally expressed genes. The opposite pattern is expected if male-biased dispersal was present.

Interestingly, in both autistic and psychopathic disorders, sleep is also affected. Accordingly, autism is associated with insomnia and lower levels of sleep [87] while psychopathic disorders tend to be associated with deeper sleep [88–90]. These patterns appear to match the predictions of sleep sacrifice being associated with altruism, but could alternatively be a consequence of anxiety in autism [91] and mental resilience in psychopathic disorders [92]. Others suggest a different continuum, where psychosis—instead of psychopathic disorders—is the opposite extreme of autism [83] and concerning parental-offspring conflict traits, similar to the ones described above for Prader–Willi and Angelman syndromes and with sleep also being affected in an identical way [93].

Some patterns of parent-of-origin gene expression have already been found for genes associated with sleep and not associated with chromosomic regions responsible for Prader–Willi and Angelman syndromes, specifically six genes in an experimental study with mouse strains [72], which suggests that genomic imprinting may be present. More research is necessary to understand if that is indeed evidence of genomic imprinting and if it follows the patterns that we propose. Additionally, genomic-wide association analysis and heritability studies show tentative evidence for a genetic component for several sleeping disorders, such as insomnia [94], obstructive sleep apnoea [95], restless leg syndrome [96], narcolepsy and essential hypersomnia [97], sleepwalking [98], sleep terrors [99], and sleep paralysis [100]. To our knowledge, the possibility that genomic imprinting is involved in any of those disorders has yet to be investigated.

## 6. Conclusion

Sleep is not usually considered to be a social behaviour. Here, we have argued that an approach that takes the social impact of sleep into consideration can offer new insights into its evolutionary drivers. We have shown how the social environment may shape an individual's sleeping pattern and also explain sexual differences in sleep requirements. Moreover, our approach also predicts the existence—and patterns—of genomic imprinting in relation to loci that underpin sleep phenotypes. By taking a new approach to the study of sleep, we have integrated our results with what is already known from the literature to present new perspectives. Further empirical work is necessary to determine if relatedness has indeed had a modulating role in the evolution of sleep. If so, then our analysis suggests that it may be crucial for understanding the evolution of sleeping patterns and associated disorders.

## Supplementary Material

Mathematical analyses and supplementary figures

## Data accessibility

This article has no additional data.
