# Supplementary material for: The social evolution of sleep: sex differences, intragenomic conflicts and clinical pathologies
Source: Proc Biol Sci. 2019 Jan 2;286(1894):20182188. doi: 10.1098/rspb.2018.2188 (PMC6367171; doi:10.1098/rspb.2018.2188)
Supplement: Mathematical analyses and supplementary figures [file rspb20182188supp1.pdf]

Electronic supplementary material for Faria et al. 2018 "The social evolution of sleep: sex differences, intragenomic conflicts and clinical pathologies"

First, we introduce a basic model for the social evolution of sleep with three analyses: 1) absence of sex differences and genes do not know their origin; 2) absence of sex differences and genes know their origin; and 3) sex differences and genes do not know their origin. And second, we introduce an illustrative model for the social evolution of sleep with two analyses: 1) sleep cannot evolve independently in females and males; and 2) sleep can evolve independently in females and males.

1. Basic model of the social evolution of sleep and inclusive fitness predictions

Natural selection favours any gene that is associated with greater individual fitness (Fisher 1930; Price 1970). Assuming vanishingly little genetic variation, this condition may be expressed using the mathematics of differential calculus: $\mathrm{d}W/\mathrm{d}g > 0$, where g is the genic value of a gene picked at random from the population and W is the relative fitness of the individual carrying this gene (Taylor 1996). We consider three scenarios (defined by the set A = {U,M,P}), concerning whether the gene's action is independent of its parent of origin (in which case the gene can be considered ignorant of its parent of origin; A = U), whether the gene's action is conditional upon it being of maternal origin (A = M), or whether the gene's action is conditional upon it being of paternal origin (A = P). We assume separate sexes (defined by the set i = {m,f}), such that a given carrier of the gene may be female (i = f) or male (i = m). Accordingly, the appropriate measure of relative fitness is a class-reproductive-value-weighted average taken across females and males, i.e. $W = ½W_\mathrm{f} + ½W_\mathrm{m}$, where W_f is the relative fitness of the female carrying the gene and W_m is the relative fitness of the male carrying the gene (Taylor 1996; Taylor & Frank 1996). The relative fitness of a female may be written as $W_\mathrm{f}(x_\mathrm{f}, y_\mathrm{f}, z_\mathrm{f})$, where x_f is the level of sleep of the focal female, y_f is the average level of sleep of the females in the focal patch, and z_f is the average level of sleep of the females in the population, with values ranging from 0 (no sleep) to 1 (sleep throughout the whole sleeping period). Similarly, the relative fitness of a male may be written as $W_\mathrm{m}(x_\mathrm{m}, y_\mathrm{m}, z_\mathrm{m})$, where x_m is the level of sleep of the focal male, y_m is the average level of sleep of the males in the focal patch, and z_m is the average level of sleep of the males in the population, with values again ranging from 0 (no sleep) to 1 (sleep throughout the whole sleeping period).

Following the approach of Taylor & Frank (1996) for a class-structured population, we may write $\mathrm{d}W/\mathrm{d}g_{\mathrm{i|A}} = ½ (\mathrm{d}W_\mathrm{f}/\mathrm{d}g_{\mathrm{f|A}}) + ½ (\mathrm{d}W_\mathrm{m}/\mathrm{d}g_{\mathrm{m|A}}) = ½ ((\partial W_\mathrm{f}/\partial x_\mathrm{f})(\mathrm{d}x_\mathrm{f}/\mathrm{d}G_\mathrm{f})(\mathrm{d}G_\mathrm{f}/\mathrm{d}g_{\mathrm{f|A}}) + (\partial W_\mathrm{f}/\partial y_\mathrm{f})(\mathrm{d}y_\mathrm{f}/\mathrm{d}G_\mathrm{f}')(\mathrm{d}G_\mathrm{f}'/\mathrm{d}g_{\mathrm{f|A}}) + (\partial W_\mathrm{f}/\partial y_\mathrm{m})(\mathrm{d}y_\mathrm{m}/\mathrm{d}G_\mathrm{m}')(\mathrm{d}G_\mathrm{m}'/\mathrm{d}g_{\mathrm{f|A}})) + ½ ((\partial W_\mathrm{m}/\partial x_\mathrm{m})(\mathrm{d}x_\mathrm{m}/\mathrm{d}G_\mathrm{m})(\mathrm{d}G_\mathrm{m}/\mathrm{d}g_{\mathrm{m|A}}) + (\partial W_\mathrm{m}/\partial y_\mathrm{m})(\mathrm{d}y_\mathrm{m}/\mathrm{d}G_\mathrm{m}')(\mathrm{d}G_\mathrm{m}'/\mathrm{d}g_{\mathrm{m|A}}) + (\partial W_\mathrm{m}/\partial y_\mathrm{f})(\mathrm{d}y_\mathrm{f}/\mathrm{d}G_\mathrm{f}')(\mathrm{d}G_\mathrm{f}'/\mathrm{d}g_{\mathrm{m|A}}))$, where: G_f is the focal female's breeding value, G_f' is the average breeding value of the females in the focal patch; $\mathrm{d}x_\mathrm{f}/\mathrm{d}G_\mathrm{f} = \mathrm{d}y_\mathrm{f}/\mathrm{d}G_\mathrm{f}' = \gamma_\mathrm{f}$ is the mapping between genotype and phenotype in the females; $\mathrm{d}G_\mathrm{f}/\mathrm{d}g_{\mathrm{f|A}} = p_{\mathrm{f|A}}$ is the consanguinity of the genic actor A in the focal female to the female herself; $\mathrm{d}G_\mathrm{f}'/\mathrm{d}g_{\mathrm{f|A}} = p_{\mathrm{ff|A}}$ is the consanguinity of the genic actor A in the focal female with a randomly-chosen female on her patch; $\mathrm{d}G_\mathrm{m}'/\mathrm{d}g_\mathrm{f} = p_{\mathrm{fm|A}}$ is the consanguinity of the genic actor A in the focal female with a randomly-chosen male on her patch; G_m is the focal male's breeding value; G_m' is the average breeding value of the males in the focal patch; $\mathrm{d}x_\mathrm{m}/\mathrm{d}G_\mathrm{m} = \mathrm{d}y_\mathrm{m}/\mathrm{d}G_\mathrm{m}' = \gamma_\mathrm{m}$ is the mapping between genotype and phenotype in the males; $\mathrm{d}G_\mathrm{m}/\mathrm{d}g_\mathrm{m} = p_{\mathrm{m|A}}$ is the consanguinity of the genic actor A in the focal male to the male himself; $\mathrm{d}G_\mathrm{f}'/\mathrm{d}g_\mathrm{m} = p_{\mathrm{mf|A}}$ is the consanguinity of the genic actor A in the focal male with a randomly-chose female on his patch; and $\mathrm{d}G_\mathrm{m}'/\mathrm{d}g_\mathrm{m} = p_{\mathrm{mm|A}}$ is the consanguinity of the genic actor A in the focal male with a randomly-chosen male on his patch. The consanguinity between a genic actor A to its carrier

is the same no matter the class of the genic actor A or the sex that we are considering and, therefore, $p_{f|A} = p_{m|A} = p$. We divide all terms of the right-side of the equation by p to get the kin-selection coefficient of relatedness (see below; Bulmer 1994).

If sleep cannot evolve independently in females and males, then $\gamma_f = 1$ and $\gamma_m = 1$. In this scenario, all derivatives are evaluated at $x_f = x_m = y_f = y_m = z_f = z_m = z$. Accordingly, natural selection favours an increase in the level of sleep in females and males if:

$$(C_f(z) + B_{ff}(z)r_{ff|A} + B_{fm}(z)r_{fm|A}) + (C_m(z) + B_{mm}(z)r_{mm|A} + B_{mf}(z)r_{mf|A}) > 0, \quad \text{(A1)}$$

where: $C_f(z) = \partial W_f/\partial x_f$; $B_{ff}(z) = \partial W_f/\partial y_f$; $B_{fm}(z) = \partial W_f/\partial y_m$; $C_m(z) = \partial W_m/\partial x_m$; $B_{mm}(z) = \partial W_m/\partial y_m$; $B_{mf}(z) = \partial W_m/\partial y_f$; $r_{ff|A} = p_{ff|A}/p$; $r_{fm|A} = p_{fm|A}/p$; $r_{mm|A} = p_{mm|A}/p$; and $r_{mf|A} = p_{mf|A}/p$. If sleep can evolve independently in females and males, then $\gamma_f = 1$ and $\gamma_m = 0$ when analysing sleep in females and $\gamma_f = 0$ and $\gamma_m = 1$ when analysing sleep in males. In this scenario, all derivatives are evaluated at $x_f = y_f = z_f$ and at $x_m = y_m = z_m$. Accordingly, natural selection favours an increase in the level of sleep in females if:

$$C_f(z_f) + B_{ff}(z_f)r_{ff|A} + B_{mf}(z_f)r_{mf|A} > 0 \quad \text{(A2)}$$

and an increase in the level of sleep in males if:

$$B_{fm}(z_m)r_{fm|A} + C_m(z_m) + B_{mm}(z_m)r_{mm|A} > 0. \quad \text{(A3)}$$

1.1 Inclusive fitness predictions when there are no sex differences and genes do not know their origin

We now assume that there are no sex differences and that genes are ignorant to their origin. Therefore, we can simplify the inequality (A1) to $C(z) + B(z)r_U > 0$, where r_U is the average relatedness between a genic actor ignorant to its origin in the focal individual and a random individual in her patch, $C(z)$ is how sleep of the focal individual impacts her own fitness, and $B(z)$ is how the sleep of the focal individual's social partners impacts the fitness of the focal individual. We define a function $J(z^*, r_U) = C(z^*) + B(z^*)r_U$, where z^* represents a sleep optimum (formally, a convergence stable strategy; Christiansen 1991; Taylor 1996). Being a sleep optimum means that the population is at its sleep equilibrium and, therefore, $J(z^*, r_U) = 0$. To be an evolutionary stable equilibrium, it also needs to be convergent stable and the condition $\partial J/\partial z^* < 0$ needs to be met. Making those assumptions, and using the chain rule of derivation, we get $dJ/dr_U = (\partial J/\partial r_U) + (\partial J/\partial z^*)(dz^*/dr_U) = 0$ and, rearranging, $dz^*/dr_U = -(\partial J/\partial r_U)/(\partial J/\partial z^*)$. Defining a function S that returns the sign (positive, negative, or zero), we obtain $S(dz^*/dr_U) = S(\partial J/\partial r_U) = S(B(z^*))$ (Pen 2000; Farrell et al. 2015). Consequently, if social partners' sleep improves the individual's fitness ($B > 0$), then higher relatedness is associated with a higher sleep optimum ($dz^*/dr_U > 0$); if social partners' sleep decreases the individual's fitness ($B < 0$), then higher relatedness is associated with a lower sleep optimum ($dz^*/dr_U < 0$); if social partners' sleep does not affect the individual's fitness (B = 0), then higher relatedness is not associated with a higher or lower sleep optimum ($dz^*/dr_U = 0$).

1.2 Inclusive fitness predictions when there are no sex differences and genes know their origin

We now assume that there are no sex differences and that genes do know their origin. Therefore, we can simplify the inequality (A1) to $C(z) + B(z)r_A > 0$, where r_A is the average relatedness between a genic actor A in the focal individual and a random individual in her patch. Following the approach from section 1.1, we get $S(dz^*/dr_A) = S(\partial J/\partial r_A) = S(B(z^*))$

(Pen 2000; Farrell et al. 2015). Therefore, 1) if the sleep of social partners improves an individual's fitness ($B > 0$), then the sleep optimum is higher for maternal-origin genes than it is for paternal-origin genes ($z_M^* > z_P^*$, where z_M^* represents a sleep optimum for the maternal-origin genes and z_P^* represents a sleep optimum for the paternal-origin genes) when relatedness is higher for the former than for the latter ($r_M > r_P$, where r_M represents the average relatedness between a maternal-origin gene in the focal individual and a random individual in her patch and r_P represents the average relatedness between a paternal-origin gene in the focal individual and a random individual in her patch), and the sleep optimum is lower for maternal-origin genes than it is for paternal-origin genes ($z_M^* < z_P^*$) when relatedness is lower for the former than for the latter ($r_M < r_P$); 2) if the sleep of social partners decreases an individual's fitness (B < 0), then the sleep optimum is lower for maternal-origin genes than it is for paternal-origin genes ($z_M^* < z_P^*$) when relatedness is higher for the former than for the latter ($r_M > r_P$), and the sleep optimum is higher for maternal-origin genes than it is for paternal-origin genes ($z_M^* > z_P^*$) when relatedness is lower for the former than for the latter ($r_M < r_P$); and 3) if the sleep of social partners does not affect an individual's fitness ($B = 0$), then the sleep optimum for maternal-origin genes is equal to that for paternal-origin genes ($z_M^* \approx z_P^*$) and relatedness does not shape the sleep optimum.

1.3 Inclusive fitness predictions when there are sex differences and genes do not know their origin

We now assume that females and males may have different relatedness values to their social partners, with the costs and benefits associated with a given sleeping schedule being the same. We also assume that genes do not know their origin. Therefore, we can simplify the inequalities (A2) and (A3) to $C_f(z_f) + B_{ff}(z_f)r_{ff|U} + B_{mf}(z_f)r_{ff|U} > 0$ and $C_m(z_m) + B_{mm}(z_m)r_{mm|U} + B_{fm}(z_m)r_{fm|U} > 0$, respectively. For simplicity, we assume that $C_f = C_m = C$ and that $B_{ff} = B_{fm} = B_{mm} = B_{mf} = B$, meaning that the inequalities become $C(z_f) + B(z_f)r_{f|U} > 0$ and $C(z_m) + B(z_m)r_{m|U} > 0$, and $r_{f|U}$ is the average relatedness for the females in their patch and $r_{m|U}$ is the average relatedness for the males in their patch for a genic actor ignorant to its origin. Following the same strategy as in section 1.1, an evolutionary stable equilibrium also needs to be convergent stable and the conditions $\partial J/\partial z_f^* < 0$ and $\partial J/\partial z_m^* < 0$ need to be met (where z_f^* represents a sleep optimum for the females and z_m^* represents a sleep optimum for the males). Using the chain rule of derivation, we get $dJ/dr_{f|U} = (\partial J/\partial r_{f|U}) + (\partial J/\partial z_f^*)(dz_f^*/dr_{f|U}) = 0$, which rearranges to $dz_f^*/dr_{f|U} = -(\partial J/\partial r_{f|U})/(\partial J/\partial z_f^*)$, and $dJ/dr_{m|U} = (\partial J/\partial r_{m|U}) + (\partial J/\partial z_m^*)(dz_m^*/dr_{m|U}) = 0$, which rearranges to $dz_m^*/dr_{m|U} = -(\partial J/\partial r_{m|U})/(\partial J/\partial z_m^*)$. Defining a function S that returns the sign (positive, negative, or zero), we obtain $S(dz_f^*/dr_{f|U}) = S(dz_m^*/dr_{m|U}) = S(\partial J/\partial r_{f|U}) = S(\partial J/\partial r_{m|U}) = S(B(z_f^*)) = S(B(z_m^*))$ (Pen 2000; Farrell et al. 2015) and the same conclusions as in section 1.1 applies.

Therefore, 1) if the sleep of social partners improves an individual's fitness ($B > 0$), then the sleep optimum is higher for females than it is for males ($z_f^* > z_m^*$) when relatedness is higher for the former than for the latter ($r_{f|U} > r_{m|U}$), and the sleep optimum is lower for females than it is for males ($z_f^* < z_m^*$) when relatedness is lower for the former than for the latter ($r_{f|U} < r_{m|U}$); 2) if the sleep of social partners decreases an individual's fitness ($B < 0$), then the sleep optimum is lower for females than it if for males ($z_f^* < z_m^*$) when relatedness is higher for the former than for the latter ($r_{f|U} > r_{m|U}$), and the sleep optimum is higher for females than it is for males ($z_f^* > z_m^*$) when relatedness is lower for the former than for the latter ($r_{f|U} < r_{m|U}$); and 3) if the sleep of social partners does not affect an individual's fitness ($B = 0$), then the sleep optimum for females is equal to that for males ($z_f^* \approx z_m^*$) and relatedness does not shape the sleep optimum.

2. Illustrative model

Life cycle – We consider an infinite diploid population divided into patches (Wright 1931) containing n_{f} females and n_{m} males, with every female mating with every male in her patch, and vice versa. During their sleeping period, females and males spent a proportion of this time sleeping – level of sleep – which is necessary for the maintenance of the organism and to cooperate successfully with social partners in their patch. This is counterbalanced by the presence of an external danger, which deleterious effects increase with the level of sleep, and by the probability of gaining mating opportunities, which decreases with the level of sleep. Specifically, a female's fecundity is:

$$f_{\mathrm{f}} = (x_{\mathrm{f}} - m)^{b_{\mathrm{f}}}\left(\frac{y_{\mathrm{f}}+y_{\mathrm{m}}}{2} - m\right)\left(1 - \frac{y_{\mathrm{f}}+y_{\mathrm{m}}}{2}\right)^{a}\left(\frac{1-c_{\mathrm{f}}x_{\mathrm{f}}}{1-c_{\mathrm{f}}y_{\mathrm{f}}}\right), \quad \text{(A4)}$$

where: m is the minimal amount of sleep that individuals require; b_{f} defines how the benefits of sleep increase throughout the night for the females (close to 0 the benefits grow exponentially, close to 1 the benefits grow linearly); a is the probability of an external danger being present in the environment; and c_{f} is the probability of gaining mating opportunities by sacrificing sleep in females. Therefore, $(x_{\mathrm{f}} - m)^{b_{\mathrm{f}}}$ defines the maintenance of the focal female's body through sleep, $(\frac{y_{\mathrm{f}}+y_{\mathrm{m}}}{2} - m)$ defines the cooperation within the group, $\left(1 - \frac{y_{\mathrm{f}}+y_{\mathrm{m}}}{2}\right)^{a}$ defines the vigilance within the group, and $(\frac{1-c_{\mathrm{f}}x_{\mathrm{f}}}{1-c_{\mathrm{f}}y_{\mathrm{f}}})$ defines the mating competition between the females in the group. Likewise, a male's fecundity is:

$$f_{\mathrm{M}} = (x_{\mathrm{m}} - m)^{b_{\mathrm{m}}}\left(\frac{y_{\mathrm{f}}+y_{\mathrm{m}}}{2} - m\right)\left(1 - \frac{y_{\mathrm{f}}+y_{\mathrm{m}}}{2}\right)^{a}\left(\frac{1-c_{\mathrm{m}}x_{\mathrm{m}}}{1-c_{\mathrm{m}}y_{\mathrm{m}}}\right), \quad \text{(A5)}$$

where b_{m} defines how the benefits of sleep increase throughout the night for the males (close to 0 the benefits grow exponentially, close to 1 the benefits grow linearly) and c_{m} is the probability of gaining mating opportunities by sacrificing sleep in males. Therefore, $(x_{\mathrm{m}} - m)^{b_{\mathrm{m}}}$ defines the maintenance of the focal male's body through sleep, and $(\frac{1-c_{\mathrm{m}}x_{\mathrm{m}}}{1-c_{\mathrm{m}}y_{\mathrm{m}}})$ defines the mating competition between the males in the group. Following mating, each female produces a large number of offspring, with an even sex-ratio, in proportion to her fecundity. Adults then die. Juveniles then form groups – or buds – of large size at random within their patch and each group either disperse to a random patch with probability d_{B} or remain in the focal patch otherwise (Haldane 1932). After budding dispersal, juveniles can still disperse individually, with females dispersing with probability d_{f} and males dispersing with probability d_{m} to a random patch or else remaining in their current patch. Following individual dispersal, n_{f} females and n_{m} males survive at random within each patch to adulthood, returning the population to the beginning of the life cycle.

Natural selection – Female relative fitness in this model is given by:

$$W_{\mathrm{f}} = f_{\mathrm{f}}\left(\frac{1-d_{\mathrm{B}}}{(1-d_{\mathrm{B}})F_{\mathrm{f}}+d_{\mathrm{B}}\bar{F}_{\mathrm{f}}} + \frac{d_{\mathrm{B}}}{\bar{F}_{\mathrm{f}}}\right), \quad \text{(A6)}$$

where: $F_{\mathrm{f}} = f_{\mathrm{f}}|_{x_f=y_f}$; and $\bar{F}_{\mathrm{f}} = f_{\mathrm{f}}|_{x_f=z_f,\ y_f=z_f,\ y_m=z_m}$. Likewise, male relative fitness in this model is given by:

$$W_{\mathrm{m}} = \frac{f_{\mathrm{m}}}{F_{\mathrm{m}}}F_{\mathrm{f}}\left(\frac{1-d_{\mathrm{B}}}{(1-d_{\mathrm{B}})F_{\mathrm{f}}+d_{\mathrm{B}}\bar{F}_{\mathrm{f}}} + \frac{d_{\mathrm{B}}}{\bar{F}_{\mathrm{f}}}\right), \quad \text{(A7)}$$

where $F_{\mathrm{m}} = f_{\mathrm{m}}|_{x_m=y_m}$. We can now use the inequalities derived in section 1 to reach the marginal fitness equations for the evolution of sleep and, consequently, to derive the optimal level of sleep for the different scenarios explored in the main text (see below).

Relatedness – The relatedness between a genic actor A in the focal female with a randomly-chosen female in her patch (including the focal female herself) is approximately given by:

$$r_{\mathrm{ff|A}} = \frac{1}{n_{\mathrm{f}}} + \frac{n_{\mathrm{f}}-1}{n_{\mathrm{f}}}(1-d_{\mathrm{f}})^2 r_{\mathrm{A}}, \tag{A8}$$

where: with probability $\frac{1}{n_{\mathrm{f}}}$ the randomly chosen female is the focal female herself, in which case relatedness is 1; and with probability $\frac{n_{\mathrm{f}}-1}{n_{\mathrm{f}}}$ is a different female, in which case they are only related if they are both locals $(1-d_{\mathrm{f}})^2$ and, if so, their relatedness is defined by the relatedness through the genic actor A (r_{A}) in the focal female. The approximation becomes exact in the limit of vanishingly weak selection. For the relatedness between a genic actor A in the focal female with a random male in her patch:

$$r_{\mathrm{fm|A}} = (1-d_{\mathrm{f}})(1-d_{\mathrm{m}})r_{\mathrm{A}}, \tag{A9}$$

and they are only related if they are both locals $(1-d_{\mathrm{f}})(1-d_{\mathrm{m}})$ and, if so, their relatedness is defined by the relatedness through the genic actor A (r_{A}) in the focal female. For the relatedness between a genic actor A in the focal male and randomly-chosen male in his patch (including the focal male himself):

$$r_{\mathrm{mm|A}} = \frac{1}{n_{\mathrm{m}}} + \frac{n_{\mathrm{m}}-1}{n_{\mathrm{m}}}(1-d_{\mathrm{m}})^2 r_{\mathrm{A}}, \tag{A10}$$

where: with probability $\frac{1}{n_{\mathrm{m}}}$ the randomly chosen male is the focal male himself, in which case relatedness is 1; and with probability $\frac{n_{\mathrm{m}}-1}{n_{\mathrm{m}}}$ is a different male, in which case they are only related if they are both locals $(1-d_{\mathrm{m}})^2$ and, if so, their relatedness is defined by the relatedness through the genic actor A (r_{A}) in the focal male. For the relatedness between a genic actor A in the focal male with a random female in his patch:

$$r_{\mathrm{mf|A}} = (1-d_{\mathrm{m}})(1-d_{\mathrm{f}})r_{\mathrm{A}}, \tag{A11}$$

and they are only related if they are both locals $(1-d_{\mathrm{m}})(1-d_{\mathrm{f}})$ and, if so, their relatedness is defined by the relatedness through the genic actor A (r_{A}) in the focal male. Note that $r_{\mathrm{mf|A}} = r_{\mathrm{fm|A}}$ and, therefore, we use $r_{\mathrm{fm|A}}$ to represent both throughout the rest of the document.

Relatedness through the genic actor A between two different juveniles born in the same patch is then given by $r_{\mathrm{A}} = p_{\mathrm{A}}'/p$, where p_{A}' is the consanguinity through the genic actor A between two individuals born in the same patch and is defined by picking the genic actor A from the focal individual and a random gene from the other individual and calculating the probability that the two are identical by descent (Bulmer 1994). Focusing upon genes ignorant of their origin (A = U) and assuming that consanguinities are at their neutral-equilibrium values, appropriate if selection is weak (Gardner et al. 2011), we write:

$$p_{\mathrm{U}}' = \frac{1}{4}\left(\frac{1}{n_{\mathrm{f}}}p + \frac{n_{\mathrm{f}}-1}{n_{\mathrm{f}}}(1-d_{\mathrm{f}})^2 p_{\mathrm{U}}'\right) + \frac{1}{4}\left(\frac{1}{n_{\mathrm{m}}}p + \frac{n_{\mathrm{m}}-1}{n_{\mathrm{m}}}(1-d_{\mathrm{m}})^2 p_{\mathrm{U}}'\right) + \frac{1}{2}(1-d_{\mathrm{f}})(1-d_{\mathrm{m}})p_{\mathrm{U}}', \tag{A12}$$

where: with probability of ¼ we may have drawn the maternal-origin genes from both individuals, in which case with probability of $\frac{1}{n_\mathrm{f}}$ they share the same mother (and they have consanguinity of p) and with probability of $\frac{n_\mathrm{f}-1}{n_\mathrm{f}}$ they have different mothers (and they will only have consanguinity if both mothers are local, giving a consanguinity of $(1-d_\mathrm{f})^2 p_\mathrm{U}'$); with probability of ¼ we may have drawn the paternal-origin genes from both individuals, in which case with probability of $\frac{1}{n_\mathrm{m}}$ they share the same father (and they have consanguinity of p) and with probability of $\frac{n_\mathrm{m}-1}{n_\mathrm{m}}$ they have different fathers (and they will only have consanguinity if both fathers are local, giving a consanguinity of $(1-d_\mathrm{m})^2 p_\mathrm{U}'$); and with probability of ½ we have drawn the maternal-origin gene from one and the paternal-origin gene from the other and they will only have consanguinity if both these parents are locals (giving a consanguinity of $(1-d_\mathrm{f})(1-d_\mathrm{m})p_\mathrm{U}'$). Rearranging, we get:

$$p_\mathrm{U}' = \frac{n_\mathrm{f}+n_m}{(1-d_\mathrm{f})^2 n_\mathrm{m}+(1-d_\mathrm{m})^2 n_\mathrm{f}+(4-d_\mathrm{f}-d_\mathrm{m})(d_\mathrm{f}+d_\mathrm{m})n_\mathrm{m}n_\mathrm{f}} p, \tag{A13}$$

and the relatedness between two random individuals born in the same patch is then given by $r_\mathrm{U} = p_\mathrm{U}'/p$ (Bulmer 1994). Rearranging, we obtain:

$$r_\mathrm{U} = \frac{n_\mathrm{f}+n_m}{(1-d_f)^2 n_\mathrm{m}+(1-d_m)^2 n_\mathrm{f}+(4-d_\mathrm{f}-d_\mathrm{m})(d_f+d_\mathrm{m})n_\mathrm{m}n_\mathrm{f}}. \tag{A14}$$

But we can also separate the consanguinity between two juveniles in their maternal- and paternal-origin components. That is:

$$p_\mathrm{U}' = \frac{1}{2}(p_\mathrm{M}' + p_\mathrm{P}'), \tag{A15}$$

and by its turn:

$$p_\mathrm{M}' = \frac{1}{2}\left(\frac{1}{n_\mathrm{f}}p + \frac{n_\mathrm{f}-1}{n_\mathrm{f}}(1-d_\mathrm{f})^2 p_\mathrm{U}'\right) + \frac{1}{2}(1-d_\mathrm{f})(1-d_\mathrm{m})p_\mathrm{U}'; \tag{A16}$$

$$p_\mathrm{P}' = \frac{1}{2}\left(\frac{1}{n_\mathrm{m}}p + \frac{n_\mathrm{m}-1}{n_\mathrm{m}}(1-d_\mathrm{m})^2 p_\mathrm{U}'\right) + \frac{1}{2}(1-d_\mathrm{f})(1-d_\mathrm{m})p_\mathrm{U}'. \tag{A17}$$

Relatedness between two random individuals in the same patch through their maternal-origin genes is then given by $r_\mathrm{M} = p_\mathrm{M}'/p$ (Bulmer 1994) and through their paternal-origin genes by $r_\mathrm{P} = p_\mathrm{P}'/p$ (Bulmer 1994). Rearranging, we obtain:

$$r_\mathrm{M} = \frac{(2-d_\mathrm{f}-d_\mathrm{m})\left(n_\mathrm{f}-d_\mathrm{m}+d_\mathrm{f}(1-n_\mathrm{f})\right)+n_\mathrm{m}(2+d_\mathrm{f}(1-d_\mathrm{m})+d_\mathrm{m}(3-d_\mathrm{m}))}{2n_\mathrm{f}(1-d_\mathrm{m})^2+2n_\mathrm{m}(1-d_f)^2+2n_f n_\mathrm{m}(4-d_\mathrm{f}-d_\mathrm{m})(d_f+d_\mathrm{m})}; \tag{A18}$$

$$r_\mathrm{P} = \frac{{d_f}^2(1-n_f)+d_\mathrm{m}(2+n_\mathrm{f}-d_\mathrm{m}(1-n_\mathrm{m})-3n_\mathrm{m})+2(n_\mathrm{f}+n_\mathrm{m})-d_\mathrm{f}(2-n_\mathrm{f}(3-d_\mathrm{m})+n_\mathrm{m}(1-d_\mathrm{m}))}{2n_\mathrm{f}(1-d_\mathrm{m})^2+2n_\mathrm{m}(1-d_f)^2+2n_f n_\mathrm{m}(4-d_\mathrm{f}-d_\mathrm{m})(d_f+d_\mathrm{m})}. \tag{A19}$$

We can replace the equations (A14; A18-19) into the equations (A8-11) to obtain the different coefficients of relatedness for genes ignorant of their origin (A = U), for maternal-origin genes (A = M), and for paternal-origin genes (A = P), respectively.

2.1 Evolution of sleep cannot evolve independently in females and males

Sentinel model – Here we explore the case where individuals can sacrifice sleep to increase the vigilance in their group but not their mating opportunities ($c_f = c_m = 0$). We assume that the benefits that females and males get throughout their sleep is similar ($b_f = b_m = b$), that sleep cannot evolve independently in females and males ($\gamma_f = 1$; $\gamma_m = 1$), and diploidy. We now can obtain the derivatives of the left side of the inequality (A1) and obtain the marginal fitness equation for the present model:

$$\frac{2b\left(2-(1-d_B)^2 r_{ff|A}+(2-d_B)d_B r_{fm|A}-r_{mm|A}\right)(1-z^*)+(2-d_B)d_B(r_{ff|A}+2r_{fm|A}+r_{mm|A})(1+a\,m-(1+a)z^*)}{4(z^*-m)(1-z^*)}=0. \quad \text{(A20)}$$

Now we can solve the equation (A20) for z^* to get the equation for the optimal level of sleep:

$$z^*=\frac{d_B(2-d_B)(1+am)\left(r_{ff|A}+2r_{fm|A}+r_{mm|A}\right)+2b(2\left(1+d_B r_{fm|A}\right)-(1-d_B)^2 r_{ff|A}-r_{fm|A}{d_B}^2-r_{mm|A})}{d_B(1+a)(2-d_B)\left(r_{ff|A}+2r_{fm|A}+r_{mm|A}\right)+2b(2\left(1+d_B r_{fm|A}\right)-(1-d_B)^2 r_{ff|A}-r_{fm|A}{d_B}^2-r_{mm|A})}. \quad \text{(A21)}$$

We can now use the equation (A21) with the values of the main text to get the Figure 1*a*, with the assumption that genes are ignorant to their origin (A = U). We can also obtain Figure S1*a* with that same equation, following the same assumption, but now using different values (see below). Similarly, we can obtain the values of sleep favoured by the maternal-origin genes (A = M) and by the paternal-origin genes (A = P) as shown in Figure 3*a* and Figure S3*a*.

Reproductive model – Here we explore the case where individuals can sacrifice sleep to gain additional mating opportunities but not to increase the vigilance in their group ($a = 0$). We assume that females and males benefit from this strategy to the same degree ($c_f = c_m = c$). We also assume that the benefits that females and males get throughout their sleep is similar ($b_f = b_m = b$), that sleep cannot evolve independently in females and males ($\gamma_f = 1$; $\gamma_m = 1$), and diploidy. Using the same approach as above, we get the following marginal fitness equation:

$$\frac{(1-cz^*)\left(d_B(2-d_B)\left(r_{ff|A}+2r_{fm|A}+r_{mm|A}\right)+2b\left(2-(1-d_B)^2 r_{ff|A}+d_B(2-d_B)r_{fm|A}-r_{mm|A}\right)\right)-2c(2-r_{ff|A}-r_{mm|A})(z^*-m)}{4(z-m)(1-cz^*)}=0. \quad \text{(A22)}$$

Now we can solve the equation (A22) for z^* to get the equation for the optimal level of sleep:

$$z^*=\frac{2cm\left(2-r_{ff|A}-r_{mm|A}\right)+d_B(2-d_B)\left(r_{ff|A}+2r_{fm|A}+r_{mm|A}\right)+2b\left(2-(1-d_B)^2 r_{ff|A}+d_B(2-d_B)r_{fm|A}-r_{mm|A}\right)}{c(2\left(2-r_{ff|A}-r_{mm|A}\right)+d_B(2-d_B)\left(r_{ff|A}+2r_{fm|A}+r_{mm|A}\right)+2b\left(2-(1-d_B)^2 r_{ff|A}+d_B(2-d_B)r_{fm|A}-r_{mm|A}\right))}. \quad \text{(A23)}$$

We can now use the equation (A23) with the values of the main text to get the Figure 1*b*, with the assumptions that genes are ignorant to their origin (A = U). We can also obtain Figure S1*b* with that same equation, following the same assumption, but now using different values (see below). As before, we can also obtain the values of sleep favoured by the maternal-origin genes (A = M) and by the paternal-origin genes (A = P) as shown in Figure 3*b* and Figure S3*b*.

2.2 Evolution of sleep can evolve independently in females and males

Sentinel model – We now assume that sleep can evolve independently in females and males. As before, we assume that individuals can sacrifice sleep to increase the vigilance in their group but not their mating opportunities ($c_f = c_m = 0$), that the benefits that females and males get throughout their sleep is similar ($b_f = b_m = b$), that genes are ignorant to their origin (A = U), and diploidy. We now focus on females' sleep ($\gamma_f = 1$; $\gamma_m = 0$). We can obtain the

derivatives of the left side of the inequality (A2) and, with that, the marginal fitness equation for the females:

$$\frac{1}{2}\left(\frac{b(1-r_{\mathrm{ff|U}}(1-d_{\mathrm{B}})^2+r_{\mathrm{fm|U}}d_{\mathrm{B}}(2-d_{\mathrm{B}})}{z_{\mathrm{f}}^*-m}+d_{\mathrm{B}}(2-d_{\mathrm{B}})(r_{\mathrm{ff|U}}+r_{\mathrm{fm|U}})(\frac{1}{z_{\mathrm{f}}^*+z_{\mathrm{m}}-2m}-\frac{a}{2-z_{\mathrm{f}}^*-z_{\mathrm{m}}})\right)=0. \quad \text{(A24)}$$

We now focus on males' sleep ($\gamma_{\mathrm{f}} = 0$; $\gamma_{\mathrm{m}} = 1$). We can obtain the derivatives of the left side of the inequality (A3) and, with that, the marginal fitness equation for the males:

$$\frac{1}{2}\left(\frac{b(1-r_{\mathrm{mm|U}})}{z_{\mathrm{m}}^*-m}+d_{\mathrm{B}}(2-d_{\mathrm{B}})(r_{\mathrm{mm|U}}+r_{\mathrm{fm|U}})(\frac{1}{z_{\mathrm{f}}+z_{\mathrm{m}}^*-2m}-\frac{a}{2-z_{\mathrm{f}}-z_{\mathrm{m}}^*})\right)=0. \quad \text{(A25)}$$

Now we can solve the system of equations (A24-25) to get the optimal solutions for both z_{f}* and z_{m}*, similar to what we did above. Those solutions can then be used to get the Figure 2*a*, using the values of the main text. Note, however, that using those values means that the equation (A24) is always positive and, as such, females are selected to sleep as much as possible. This result needs to be incorporated into the equation (A25) when trying to find the males' optimal level of sleep. When doing so, there are two solutions but only one makes sense given the assumptions of the model. A similar pattern is present when using the values of Figure S2*a*. However, after a certain value of male dispersal, the equation is no longer always positive, meaning that we can simply use the solutions of the system of equations (A24-25) to represent the optimal level of sleep for both females and males.

Reproductive model – As in the previous section, here we assume that sleep can evolve independently in females and males, but now we assume that males are the only ones that can sacrifice sleep to increase their mating opportunities ($c_{\mathrm{f}} = 0$) and that individuals do not sacrifice sleep to increase the vigilance in their group ($a = 0$). Once again, we assume that the benefits that females and males get throughout their sleep is similar ($b_{\mathrm{f}} = b_{\mathrm{m}} = b$), that genes are ignorant to their origin (A = U), and diploidy. We now focus on females' sleep ($\gamma_{\mathrm{f}} = 1$; $\gamma_{\mathrm{m}} = 0$). We can obtain the derivatives of the left side of the inequality (A2) and, with that, the marginal fitness equation for the females:

$$\frac{1}{2}\left(\frac{b(1-r_{\mathrm{ff|U}}(1-d_{\mathrm{B}})^2+r_{\mathrm{fm|U}}d_{\mathrm{B}}(2-d_{\mathrm{B}})}{z_{\mathrm{f}}^*-m}+\frac{d_{\mathrm{B}}(2-d_{\mathrm{B}})(r_{\mathrm{ff|U}}+r_{\mathrm{fm|U}})}{z_{\mathrm{f}}^*+z_{\mathrm{m}}-2m}\right)=0. \quad \text{(A26)}$$

We now focus on males' sleep ($\gamma_{\mathrm{f}} = 0$; $\gamma_{\mathrm{m}} = 1$). We can obtain the derivatives of the left side of the inequality (A3) and, with that, the marginal fitness equation for the males:

$$\frac{1}{2}\left(\frac{b(1-r_{\mathrm{mm|U}})}{z_{\mathrm{m}}^*-m}+\frac{d_{\mathrm{B}}(2-d_{\mathrm{B}})(r_{\mathrm{mm|U}}+r_{\mathrm{fm|U}})}{z_{\mathrm{f}}+z_{\mathrm{m}}^*-2m}-\frac{c_{\mathrm{m}}(1-r_{\mathrm{mm|U}})}{1-c_{\mathrm{m}}z_{\mathrm{m}}^*}\right)=0. \quad \text{(A27)}$$

Note that equation (A26) is always positive under the assumptions of the model. Therefore, we can simply assume that females are selected to not sacrifice any sleep. Incorporating such assumptions into equation (A27) means that two solutions are possible for z_{m}*. Only one makes sense given the assumptions of the model and, therefore, we can use the values of the main text to get Figure 2*b* and, using different values (see below), Figure S2*b* to represent the males' optimal level of sleep.

References

Blumberg, M. S. 2015. Developing sensorimotor systems in our sleep. Curr. Dir. Psychol. Sci. 24:32–37.

Born, J., B. Rasch, and S. Gais. 2006. Sleep to remember. Neuroscientist 12:410–424.

Bulmer, M. G. 1994. Theoretical evolutionary ecology. Sunderland, MA: Sinauer Associates.

Christiansen, F. B. 1991. On conditions for evolutionary stability for a continuously varying character. Am. Nat. 138:37–50.

Diekelmann, S., and J. Born. 2010. The memory function of sleep. Nat. Rev. Neurosci. 11:114–126.

Bridi, M. C. D., S. J. Aton, J. Seibt, L. Renouard, T. Coleman, and M. G. Frank. 2015. Rapid eye movement sleep promotes cortical plasticity in the developing brain. Sci. Adv. 1:e1500105–e1500105.

Farrell, E. J., F. Úbeda, and A. Gardner. 2015. Intragenomic conflict over dispersal. Am. Nat. 186:E61–E71.

Field, J. M., and M. B. Bonsall. 2018. The evolution of sleep is inevitable in a periodic world. PLoS One 13:e0201615.

Fisher, R. A. 1930. The genetical theory of natural selection. Oxford, UK: Claredon Press.

Frank, M., N. Issa, and M. Stryker. 2001. Sleep enhances plasticity in the developing visual cortex. Neuron 30:275–287.

Gardner, A., S. A. West, and G. Wild. 2011. The genetical theory of kin selection. J. Evol. Biol. 24:1020–1043.

Herculano-Houzel, S. 2015. Decreasing sleep requirement with increasing numbers of neurons as a driver for bigger brains and bodies in mammalian evolution. Proc. R. Soc. B 282:20151853.

Jenkins, J. G., and K. M. Dallenbach. 1924. Obliviscence during sleep and waking. Am. J. Psychol. 35:605.

Karni, A., D. Tanne, B. Rubenstein, J. Askenasy, and D. Sagi. 1994. Dependence on REM sleep of overnight improvement of a perceptual skill. Science 265:679–682.

Maquet, P. 2001. The role of sleep in learning and memory. Science 294:1048–1052.

Meddis, R. 1975. On the function of sleep. Anim. Behav. 23:676–691.

Pen, I. 2000. Reproductive effort in viscous populations. Evolution 54:293–297.

Price, G. R. 1970. Selection and covariance. Nature 227:520-521.

Roffwarg, H. P., J. N. Muzio, and W. C. Dement. 1966. Ontogenetic development of the

human sleep-dream cycle. Science 152:604–619.

Schmidt, M. H. 2014. The energy allocation function of sleep: a unifying theory of sleep, torpor, and continuous wakefulness. Neurosci. Biobehav. Rev. 47:122–153.

Shaffery, J. P., C. M. Sinton, G. Bissette, H. P. Roffwarg, and G. A. Marks. 2002. Rapid eye movement sleep deprivation modifies expression of long-term potentiation in visual cortex of immature rats. 110:431–443.

Siegel, J. M. 2009. Sleep viewed as a state of adaptive inactivity. Nat. Rev. Neurosci. 10:747–753.

St-Onge, M. P. 2013. The role of sleep duration in the regulation of energy balance: effects on energy intakes and expenditure. J Clin Sleep Med 9:73–80.

Stickgold, R. 2005. Sleep-dependent memory consolidation. Nature 437:1272–1278.

Taylor, P. D. 1996. Inclusive fitness arguments in genetic models of behaviour. J. Math. Biol. 34:654–674.

Taylor, P. D., and S. A. Frank. 1996. How to make a kin selection model. J. Theor. Biol. 180:27–37.

Tononi, G., and C. Cirelli. 2003. Sleep and synaptic homeostasis: a hypothesis. Brain Res. Bull. 62:143–150.

Tononi, G., and C. Cirelli. 2014. Sleep and the price of plasticity: from synaptic and cellular homeostasis to memory consolidation and integration. Neuron 81:12–34.

Tononi, G., and C. Cirelli. 2006. Sleep function and synaptic homeostasis. Sleep Med. Rev. 10:49–62.

Wright, S. 1931. Evolution in mendelian populations. Genetics 97–159.

Xie, L., H. Kang, Q. Xu, M. J. Chen, Y. Liao, M. Thiyagarajan, J. O'Donnell, D. J. Christensen, C. Nicholson, J. J. Iliff, T. Takano, R. Deane, and M. Nedergaard. 2013. Sleep drives metabolite clearance from the adult brain. Science 342:373–377.

Table S1. The different hypothesized functions for sleep.

Hypothesis	Definition	References
Energy allocation	Organisms are selected to allocate energy to basic functions - growth, maintenance, reproduction - in a manner that maximizes energy use. During the waking period, organisms are selected to downregulate costly biological activities, such as the ones that allow the maintanance of the tissues. Those same activities are then upregulated during sleep, a period where energy is not being used by the individual in other activities, such as foraging or collecting environmental information.	St-Onge 2013; Schmidt 2014.
Adaptive inactivity	Depending on their ecology, organisms will have periods of time where none of their biological requirements can be satisfied due to biotic and abiotic environmental factors. Organisms are selected to maximize the efficiency of their behaviour, therefore being selected to reduce energy use when activity can be costly and not beneficial.	Meddis 1975; Siegel 2009; Field and Bonsall 2018.
Metabolic clearance	During the waking period, individuals accumulate metabolites in the interstitial space of their cerebral cortex as a result of their normal activity. Those metabolites are then cleared away during sleep.	Xie et al 2013; Herculano-Houzel 2015.
Maturation	Sleep allows for the development of the central nervous and sensorimotor systems and their correspondent functions.	Roffwarg et al 1966; Frank et al 2001; Shaffery et al 2002; Blumberg 2015; Bridi et al 2015.
Memory consolidation	Sleep promotes consolidation of memories acquired during the waking state into a network of long-term memories.	Jenkins and Dallenbach 1924; Karni et al 1994; Maquet 2001; Stickgold 2005; Born et al 2006; Diekelmann and Born 2010.
Synaptic homeostasis	Sleep normalizes synaptic activity to normal levels after a waking period where synapses are being triggered by learning and environmental stimulus, therefore restoring neuronal selectivity and the ability to learn new memories.	Tononi and Cirelli 2003; 2006; 2014.

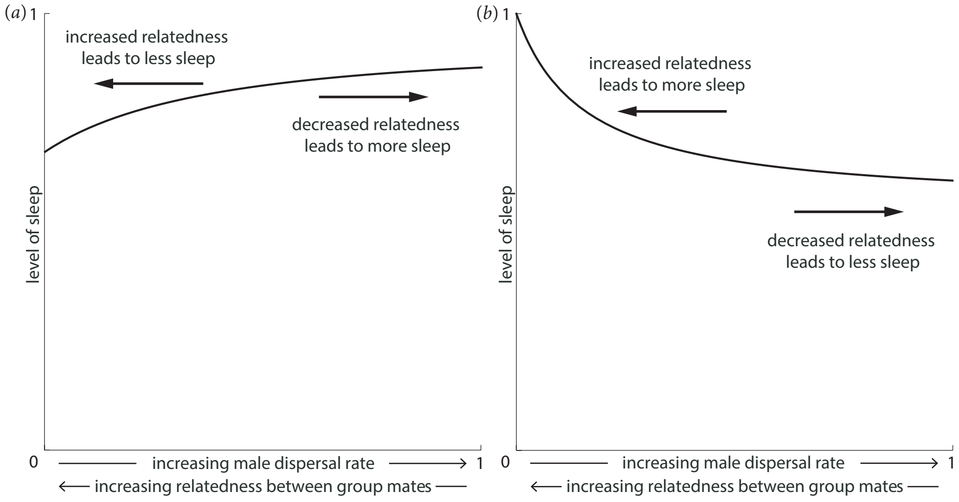

Figure S1. How much an individual should sleep depends on the relatedness between the individuals in a group. When individuals sacrifice sleep in order to remain alert to dangers which may befall the group (*a*), individuals sacrifice more sleep when relatedness is higher, which is the case when male dispersal is lower. When individuals sacrifice sleep in order to gain an advantage over their mate competitors (*b*), individuals sacrifice more sleep when relatedness is lower, which is the case when male dispersal is higher. The following parameter values were used for both panels: female dispersal rate $d_f = 0$; budding dispersal rate $d_B = 1$; number of adult females $n_f = 4$; number of adult males $n_m = 4$; minimum level of sleep $m = 0.05$; and benefits of sleeping throughout the night $b_f = b_m = 1$. Additionally, in (*a*) the level of a threat is $a = 1$ and the mating opportunities that females and males can obtain through sleep sacrifice is $c_f = c_m = 0$, while in (*b*) the level of a threat is $a = 0$ and the mating opportunities that females and males can obtain through sleep sacrifice is $c_f = c_m = 1$. Here, we consider male-biased dispersal.

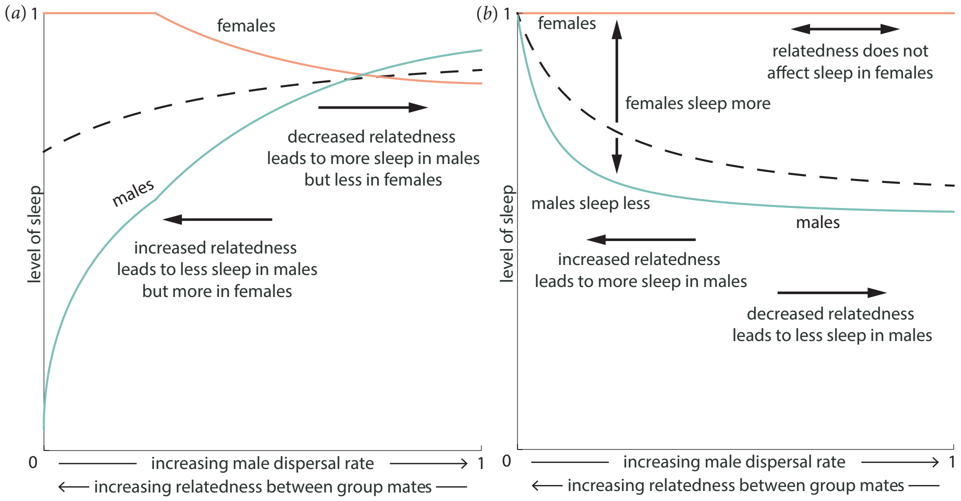

Figure S2. Females and males may be favoured to have different sleeping levels. Given that females are more related to their social partners than males, females favour less sleep when (*a*) the sleep sacrifice is being used to protect the group against threats. When (*b*) sleep sacrifice is being used to increase male reproductive success, females do not favour any sleep sacrifice, with males being the only ones to sacrifice sleep to gain an advantage over their mate competitors. Dashed line represents the favoured level of sleep when this is constrained to be the same for females and males. The following parameter values were used for both panels: female dispersal rate $d_f = 0$; budding dispersal rate $d_B = 1$; number of adult females $n_f = 4$; number of adult males $n_m = 4$; minimum level of sleep $m = 0.05$; and benefits of sleeping throughout the night $b_f = b_m = 1$. Additionally, in (*a*) the level of a threat is $a = 1$ and the mating opportunities that females and males can obtain through sleep sacrifice is $c_f = c_m = 0$, while in (*b*) the level of a threat is $a = 0$ and the mating opportunities that females and males can obtain through sleep sacrifice is $c_f = 0$ and $c_m = 1$, respectively. Here, we consider male-biased dispersal.

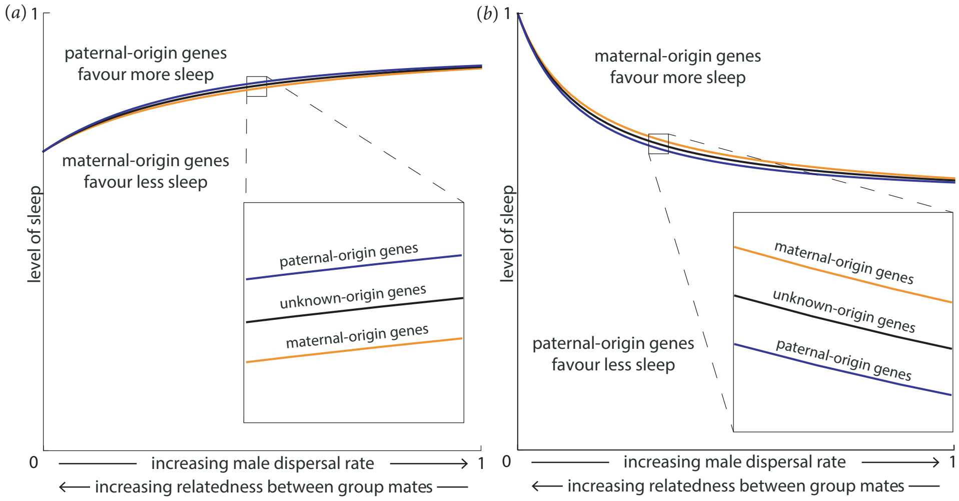

Figure S3. Maternal- and paternal-origin genes disagree regarding how much the individual should sleep. Maternal-origin genes (orange) and paternal-origin genes (blue) will disagree on how much an individual should sleep, which depends upon whether individuals are sacrificing sleep to (*a*) protect the group against threats or (*b*) gain an advantage over their mate competitors (with black being the level favoured by a gene ignorant of its origin). Specifically, given that relatedness is higher for maternal-origin genes, maternal-origin genes favour less sleep and paternal-origin genes more sleep if sleep is selfish (*a*). In constrast, if sleep is altruistic, then maternal-origin genes favour more sleep and paternal-origin genes less sleep (*b*). The following parameter values were used for both panels: female dispersal rate d_f = 0; budding dispersal rate d_B = 1; number of adult females n_f = 4; number of adult males n_m = 4; minimum level of sleep m = 0.05; and benefits of sleeping throughout the night $b_f = b_m$ = 1. Additionally, in (*a*) the level of a threat is a = 1 and the mating opportunities that females and males can obtain through sleep sacrifice is $c_f = c_m$ = 0, while in (*b*) the level of a threat is a = 0 and the mating opportunities that females and males can obtain through sleep sacrifice is $c_f = c_m$ = 1. Here, we consider male-biased dispersal.

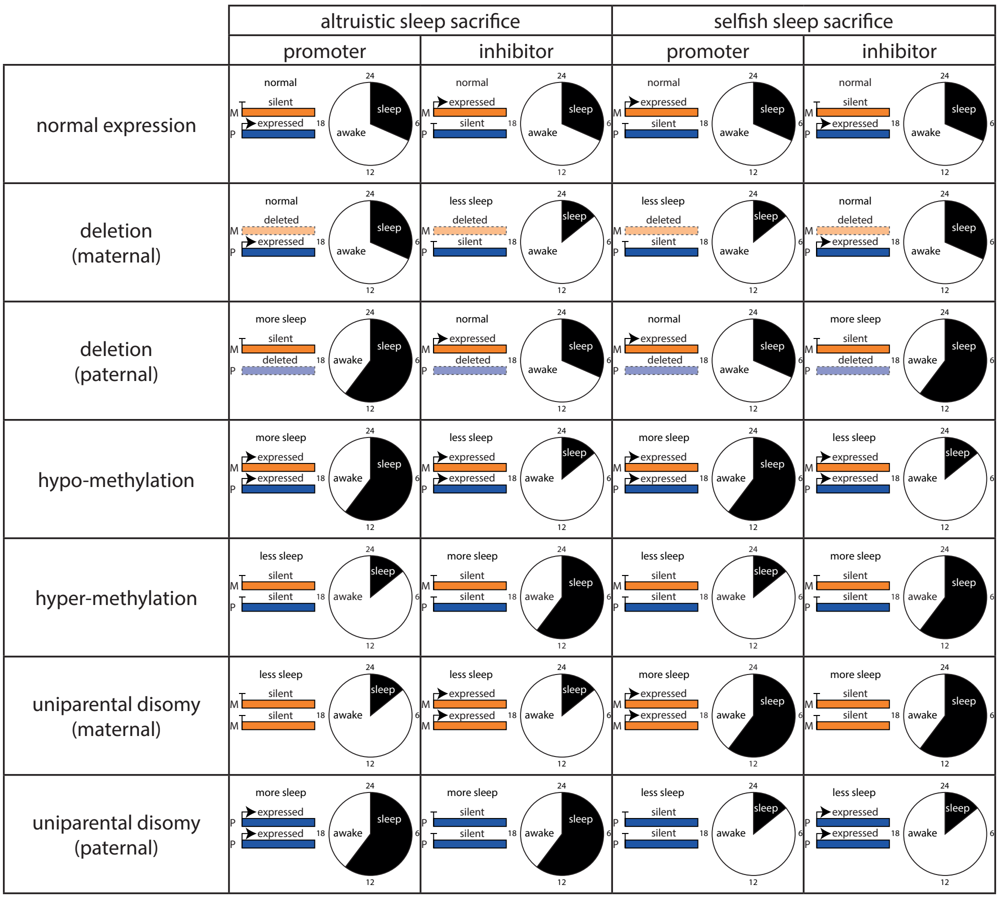

Figure S4. Genomic imprinting of genes responsible for level of sleep and the effects of possible disruptions. Predictions as to which gene is expressed and which gene is silent – maternal-origin gene (M, orange) or paternal-origin genes (P, blue) – when individuals are sacrificing sleep to protect the group against threats (altruism) or to gain an advantage over their mate competitors (selfishness). We consider an example for a gene that promotes sleep (promoter) and an example for a gene that inhibits sleep (inhibitor). In both cases, we assume male-biased dispersal. Note that for simplicity we assume methylation is associated with gene silencing, as is usually the case in mammals (Bird 2002). In cases where methylation is associated with gene activation the outcome for hypo-methylation is expected to be that shown here for hyper-methylation, and vice versa.
